# Faster onset of action with topical levocabastine than with oral cetirizine

**DOI:** 10.1155/S0962935195000779

**Published:** 1995-05

**Authors:** Michel A. Drouin, William H. Yang, Frederick Horak

**Affiliations:** 1 Section of Allergy and Clinical Immunology Department of Medicine Ottawa Civic Hospital University of Ottawa Ottawa Canada; 2 ENT Clinic University of Vienna Vienna Austria

## Abstract

This international multicentre, open-label, parallel-group trial was undertaken to compare the therapeutic efficacy and tolerability of topical levocabastine and oral cetirizine in patients with perennial allergic rhinoconjunctivitis, with particular reference to the comparative onset of action of the two drugs. A total of 207 patients were randomized to receive either levocabastine nasal spray (0.5 mg/ml, two sprays in each nostril twice daily) plus levocabastine eye drops as required (0.5 mg/ml, one drop in each eye twice daily p.r.n.) or cetirizine orally (10 mg once daily) with a treatment duration of 2 weeks. Onset of action was found to be significantly more rapid with levocabastine than with cetirizine for both nasal and ocular symptoms (p < 0.001). Within 15 min of study drug administration, 36% of levocabastine-treated patients reported relief from nasal symptoms and 32% relief from ocular symptoms compared with 10% and 17% of patients on cetirizine, respectively. At 1 h, the percentages of patients reporting relief were 76% and 38% for nasal symptoms, and 81% and 48% for ocular symptoms in the levocabastine and cetirizine treatment groups, respectively. At 8 h there were no differences between the two treatments. Overall therapeutic efficacy was found to be comparable in the two treatment groups over the 2-week study period with no significant intergroup differences in symptom severity or global therapeutic efficacy. Both drugs were well tolerated with no significant differences in the incidence or type of adverse reactions between the two groups. In conclusion, levocabastine eye drops and nasal spray are as effective and well tolerated as oral cetirizine for the treatment of perennial allergic rhinoconjunctivitis with the advantage of a significantly faster onset of action for both nasal and ocular symptoms.


					Calendar
Calendar
1996
18 January 1996
Inflammation Research Association:
Ion Channel Modulation as a
Drug Discovery Target
in Inflammatory Disease,
New York, NY, USA.
Contact: Dr Barry M. Weichman,
President, IRA,
Wyeth-Ayerst Research, CN 8000,
Princeton, NJ 08543, USA.
Fax: (+ 1) 908 274 4738.
18-19 January 1996
Perspectives in Pain Therapy:
New Strategies for the
Development of Analgesics,
Paris, France.
Contact: Institut Pasteur
Euroconferences,
Centre d'Inforrnation Scientifique,
28, rue du Docteur Roux,
75015 Paris, France.
Fax: (+33) (1) 40 61 34 05.
21-26 April 1996
8th APLAR Congress of Rheumatology,
Melbourne, Australia.
Contact: 8th Asia Pacific League
Against Rheumatism Congress,
PO Box 29, Parkville,
3052 Victoria, Australia.
Fax: (+61) (3) 387 3120.
20-23 May 1996
United States FDA Medical
Device Update: Design Controls,
GMP Requirements and
Marketing Clearance,
Paris, France.
Contact: Zena Barrick,
Medical Device Technology,
Advanstar House, Park West,
Sealand Road, Chester CH1 4RN, UK.
Fax: (+ 44) (0) 1244 370 011.
31 May-3 June 1996
26th Scandinavian Congress
of Rheumatology,
Reykjavik, Iceland.
Contact: Travel and Congress Bureau,
Feroaskrifstofa Islands,
Iceland Tourist Bureau, Skogarhlio 18,
101 Reykjavik, Iceland.
Fax: (+354) 625895/5625895.
4-7 September 1996
2nd International Congress on
Phagocytes: Biology, Physiology,
Pathology and Pharmacotherapeutics,
Pavia, Italy.
Contact: Prof. Giovanni Ricevuti,
Institute of Medical Therapy,
OSM- 27100, Pavia, Italy.
Fax: (+ 39) 382 526341.
22-27 September 1996
ICP '96
10th International Conference
on Postaglandins and
Related Compounds,
Vienna, Austria.
Contact: ICOS Congress
Organisation Service Ges.m.b.H.,
Johannesgasse 14
A-1010 Vienna, Austria.
Fax: (+43) (1) 512 80 91 80.
27-31 October 1996
New Drugs for Inflammatory,
Allergic, and Immunologic Disease:
A Symposium featured at the
8th Intemational Conference on
Inflammation Research,
Hershey, PA, USA.
Contact: Dr JB Summers,
Abbot Laboratories,
100 Abbott Park Road,
D47J AP10, Abbott Park,
It 60064, USA.
Fax: (+ 1) 708 938 5034.
1997
16-20 November 1997
The 3rd World Congress
on Inflammation,
Tokyo, Japan.
Contact: The Secretariat,
c/o PMSI Japan Ltd,
Royal Bldg 12-8 Nibancho,
Chiyoda-ku, Tokyo 102, Japan.
Fax: (+ 81) 3 5275 6994.
NOTICE FOR ADVERTISERS
To advertise in this, or any other journal from
Rapid Science Publishers
please contact Helen Dixon or Isobel Fox
Rapid Science Publishers
Middlesex House, 34-42 Cleveland Street
London WlP 6LB, UK
Tel: (+44) (0)171 323 0323; Fax: (+ 44) (0)171 436 9034
Other services available include:
Reprints of key papers
Symposium abstracts and proceedings
Proceedings and review supplements
(C) 1995 Rapid Science Publishers Mediators of Inflammation Vol 4 1995 457

				

